# Understanding Health Care Social Media Use From Different Stakeholder Perspectives: A Content Analysis of an Online Health Community

**DOI:** 10.2196/jmir.7087

**Published:** 2017-04-07

**Authors:** Yingjie Lu, Yang Wu, Jingfang Liu, Jia Li, Pengzhu Zhang

**Affiliations:** ^1^ School of Economics and Management Beijing University of Chemical Technology Beijing China; ^2^ School of Management Shanghai University Shanghai China; ^3^ School of Business East China University of Science and Technology Shanghai China; ^4^ Antai College of Economics and Management Shanghai Jiao Tong University Shanghai China

**Keywords:** health care social media, stakeholder analysis, topic analysis, sentiment analysis

## Abstract

**Background:**

Health care social media used for health information exchange and emotional communication involves different types of users, including patients, caregivers, and health professionals. However, it is difficult to identify different stakeholders because user identification data are lacking due to privacy protection and proprietary interests. Therefore, identifying the concerns of different stakeholders and how they use health care social media when confronted with huge amounts of health-related messages posted by users is a critical problem.

**Objective:**

We aimed to develop a new content analysis method using text mining techniques applied in health care social media to (1) identify different health care stakeholders, (2) determine hot topics of concern, and (3) measure sentiment expression by different stakeholders.

**Methods:**

We collected 138,161 messages posted by 39,606 members in lung cancer, diabetes, and breast cancer forums in the online community MedHelp.org over 10 years (January 2007 to October 2016) as experimental data. We used text mining techniques to process text data to identify different stakeholders and determine health-related hot topics, and then analyzed sentiment expression.

**Results:**

We identified 3 significantly different stakeholder groups using expectation maximization clustering (3 performance metrics: Rand=0.802, Jaccard=0.393, Fowlkes-Mallows=0.537; *P*<.001), in which patients (24,429/39,606, 61.68%) and caregivers (12,232/39,606, 30.88%) represented the majority of the population, in contrast to specialists (2945/39,606, 7.43%). We identified 5 significantly different health-related topics: symptom, examination, drug, procedure, and complication (Rand=0.783, Jaccard=0.369, Fowlkes-Mallows=0.495; *P*<.001). Patients were concerned most about symptom topics related to lung cancer (536/1657, 32.34%), drug topics related to diabetes (1883/5904, 31.89%), and examination topics related to breast cancer (8728/23,934, 36.47%). By comparison, caregivers were more concerned about drug topics related to lung cancer (300/2721, 11.03% vs 109/1657, 6.58%), procedure topics related to breast cancer (3952/13,954, 28.32% vs 5822/23,934, 24.33%), and complication topics (4449/25,701, 17.31% vs 4070/31,495, 12.92%). In addition, patients (9040/36,081, 25.05%) were more likely than caregivers (2659/18,470, 14.39%) and specialists (17,943/83,610, 21.46%) to express their emotions. However, patients’ sentiment intensity score (2.46) was lower than those of caregivers (4.66) and specialists (5.14). In particular, for caregivers, negative sentiment scores were higher than positive scores (2.56 vs 2.18), with the opposite among specialists (2.62 vs 2.46). Overall, the proportion of negative messages was greater than that of positive messages related to symptom, complication, and examination. The pattern was opposite for drug and procedure topics. A trend analysis showed that patients and caregivers gradually changed their emotional state in a positive direction.

**Conclusions:**

The hot topics of interest and sentiment expression differed significantly among different stakeholders in different disease forums. These findings could help improve social media services to facilitate diverse stakeholder engagement for health information sharing and social interaction more effectively.

## Introduction

People are increasingly turning to the Internet for health information. Some social media services, such as Wikipedia, Facebook, online forums, and message boards, are increasingly being used to obtain health information and share health care experiences [1,2]. A Pew survey showed that 61% of American adults looked online for health information and that 41% of this group had read about someone else’s medical experiences posted in social media. More than half thought the information online affected a decision about how to treat an illness or condition [3].

There are some reasons why patients and their caregivers turn to social media for health care information. First, they feel that doctors are too busy to answer their questions [4]; many doctors only tell their patients how to take a treatment but do not take time to fully explain the detailed reasons [5]. Second, health care social media thus allows users to learn more about their health problems through the use of social support and make decisions about treatment options [6], especially for those with chronic diseases [7]. Third, convenience and anonymity [4] are also important reasons why patients and their caregivers turn to social media, where they feel less embarrassed about asking online experts or communicating with other online members about their health conditions [8].

In addition to patients and their caregivers, other health care stakeholders are involved in health care social media, including doctors, physicians, nurses, aides, social workers, spiritual caregivers, counselors, therapists, and volunteers [9-17]. Some medical experts like to communicate with social media users and offer them professional medical knowledge. In addition, some fellow patients are willing to share their experiences with treatment, which are considered valuable for patients with newly diagnosed conditions. These individuals devote their time to helping other patients get answers in their time of need. These information providers are recognized as health specialists and make great contributions to health care social media.

Health care social media is not only used to exchange health information [18-20], but also provides a platform for emotional communication [21-23]. Some emotional expressions, such as venting, sending positive energy, and showing compassion or empathy, can help patients with serious diseases to cope. It is important especially for patients with cancer and chronic illnesses to receive emotional support and encouragement from fellow patients [24]. A study indicated that patients with low-survival-rate diseases have more emotional communication than those with high-survival-rate diseases [25]. Another study found that an estimated 75% to 85% of patients engaged in health care social media changed their sentiment in a positive direction through online interactions with other users.

Thus, different types of members discuss topics they are concerned about and share their feelings in health care social media. Understanding the concerns of different stakeholders and how they use health care social media is very meaningful work. Such information can enable users, especially newcomers, to obtain a sense of what social media is, help them quickly find the issues they are concerned about, increase their involvement more easily, and obtain the resources they need for health self-education, self-caring, and self-management. For health care social media sites, a better understanding of user online behaviors could help webmasters provide humanized supporting functions to facilitate diverse stakeholder engagement. For researchers in the field of health care, such studies could clearly summarize the behavior characteristics of different stakeholders and provide advice and guidance for health care social media.

However, given the tremendous amounts of health-related messages posted by social media users, it is difficult and time consuming to employ traditional statistical approaches to find valuable information. Moreover, social media users are usually reluctant to offer personal information due to privacy concerns, and user identification data are usually proprietary and hence not easily accessible. To address these problems, text mining techniques have recently been applied to health social media in some studies [26] to shed light on the demographics of health-related social media users by extracting keywords and other key attributes. However, clearly identifying different health care stakeholders is still a problem that needs to be solved. We therefore proposed a novel framework using text mining techniques to comprehensively analyze the content of an online health community from the perspectives of diverse stakeholders. We used a computational social science approach to process the large amount of text data for stakeholder analysis, topic analysis, and sentiment analysis. This approach allowed us to determine the characteristics of different stakeholders involved in health care social media to facilitate an understanding of health care social media use.

## Methods

### Data Collection

We chose the online health community MedHelp.org (MedHelp International, San Francisco, CA, USA) as our data source. This site is one of the most popular health care social media platforms. It consists of more than 170 discussion boards about different diseases. Since the site opened in 1994, millions of threads have been posted in the community. It also attracts over one million visitors every month.

We selected representative disease forums. Lung cancer and breast cancer are common cancers with high mortality, and some studies have shown that both cancers are among the most common cancers that Internet users seek information about [27]. Diabetes, as a chronic disease, is also among the most frequently discussed diseases in health care social media. Therefore, we chose lung cancer, breast cancer, and diabetes as our research subjects. We obtained all the webpages from these 3 disease forums by using webcrawler software Offline Explorer 6.8 (MetaProducts Systems), and then we parsed the pages to extract available messages and stored the messages in a database. Next, we filtered out noisy and unreliable data by text preprocessing. We finally collected 138,161 messages posted by 39,606 members in these 3 disease forums in the 10 years from January 2007 to October 2016 as our experimental data. Table 1 shows the statistical results.

**Table 1:** Data collection statistics (January 2007 to October 2016) from 3 disease forums on MedHelp.org.

Disease type	No. of messages	No. of members	Messages per member
Lung cancer	5317	2416	2.20
Diabetes	35,193	11,571	3.04
Breast cancer	97,651	25,619	3.81

We only used messages that were open to the public. We never used any user identification data. Personal information such as name, age, and other demographics was not used or reported as part of the results of the study. Therefore, this study did not raise any ethical or legal concerns.

### Experiment Design

**Figure 1:**
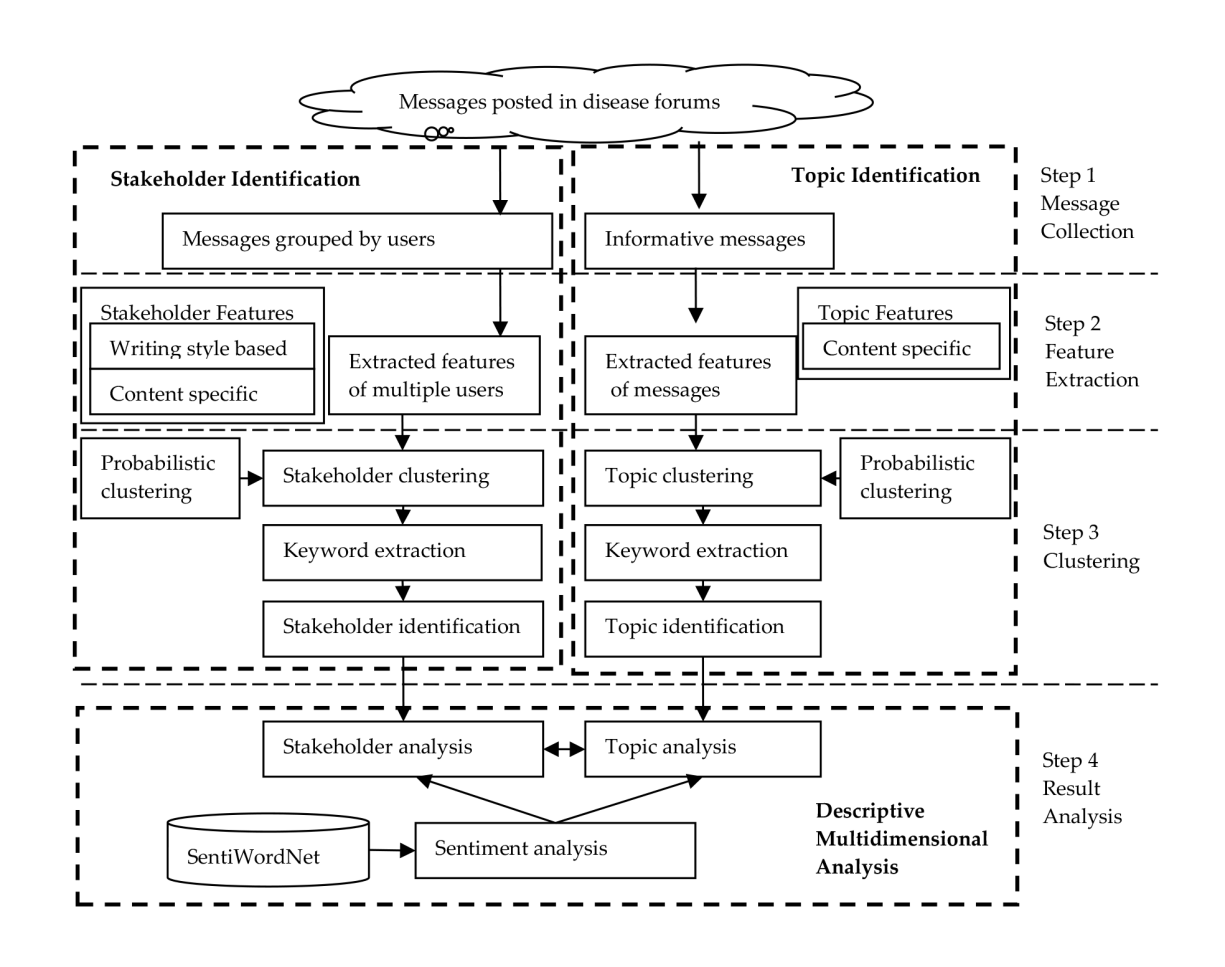
Research framework.

In this paper, we propose a novel framework using text mining techniques to comprehensively analyze the content of the messages posted in 3 disease forums. The framework consists of 3 parts: stakeholder identification, topic identification, and sentiment analysis, as Figure 1 shows.

First, to distinguish different stakeholders engaged in the online health community, we based stakeholder analysis on text mining techniques to identify different user groups. Users were automatically partitioned into different groups based on the similarity of their posts. An unsupervised approach such as a clustering technique was applied to stakeholder identification. Then, to better understand the concerns of different stakeholders, we based topic analysis on text mining techniques to identify health-related hot topics. Next, we analyzed sentiment to assess the valence and intensity of the messages posted by different stakeholders, as the emotional support and encouragement offered by community members are also an important component of online communication.

#### Stakeholder Identification

Stakeholder identification included the following 4 steps: (1) message collection, (2) feature extraction, (3) probabilistic clustering, and (4) keyword extraction and stakeholder identification.

For message collection, we first collected all messages posted in the 3 disease forums, and then merged all the messages posted by the same user into one.

For feature extraction, we used a comprehensive textual feature representation encompassing writing style-based features and content-specific features for authorship analysis [28,29]. Writing style-based features were the number of messages, average number of sentences per message, and frequency of words per message. Content-specific features were word *n*-grams, medical domain-specific terminologies, and kinship terminologies. The *n*-grams referred to word unigrams, bigrams, and trigrams and represented important keywords and phrases in the messages. Medical domain-specific terminologies were helpful in distinguishing diverse stakeholders, as some studies have shown that patients and their caregivers used more lay concepts and fewer professional terminologies than health professionals do [30-33]. We selected Medical Subject Headings (MeSH), a controlled thesaurus, to extract health-related terminologies. Family members of patients serving as primary caregivers usually used kinship terms to refer to the patients they cared for in their posts, and therefore we selected kinship terminologies as part of the content-specific features to distinguish caregivers from other stakeholders. Kinship terminologies in this study were mainly identified by one Unified Medical Language System (UMLS) semantic type, family group, which has been used in previous studies to identify family member concepts [34].

For probabilistic clustering, without a priori knowledge of the number of user groups and their specific characteristics, we chose a probabilistic clustering that leveraged the expectation maximization algorithm among various clustering techniques for stakeholder identification [35]. We found the optimal number of clusters by cross-validating different numbers of clusters. We then used 3 metrics to evaluate the clustering results: the Rand index, Jaccard similarity coefficient, and the Fowlkes-Mallows (FM) index, which we used in our previous study [36]. The clustering result was a probabilistic distribution of instances of belonging to each stakeholder cluster. We labeled each user with a group number according to the cluster with the highest probability assignment.

For keyword extraction, the keywords were terms that best distinguished the corresponding user group from other groups. We extracted these keywords from the messages posted by each user group according to their scores. Supposing all resultant clusters were C_1_,C_2_...C_N_, then for each *n*-gram (unigram, bigram, and trigram) term *w* in a cluster C_i_, its score was calculated as f(*w*, C_i_) × log (N/|{C_j_|f(*w*, C_j_) ≥ f(*w*, C_i_), *j*=1,2...N}|) (equation 1), where f(*w*, C_i_) is the frequency of *w* in cluster C_j_, and |{C_j_|f(*w*, C_j_) ≥ f(*w*, C_i_), *j*=1,2...N}| is the total number of clusters with a frequency of term *w* greater than or equal to the term *w* frequency of the cluster evaluated. We ranked keywords with high scores, from which we could infer the stakeholder identification.

#### Topic Identification

We identified topics in a manner similar to identifying stakeholders, by the following steps: message collection, feature extraction, probabilistic clustering, keyword extraction, and topic identification.

For message collection, we only chose informational messages involving health conditions, online reviews of particular drugs or medical treatments, and so on. We filtered out messages containing no informational support but only offering emotional support and other spam messages.

For feature extraction, the features used in topic identification included 2 parts: word *n*-grams and domain-specific terminologies. Domain-specific terminologies were from the UMLS Metathesaurus. The UMLS Metathesaurus as the world’s largest repository of biomedical concepts, consisting of 1.7 million biomedical concepts, where each concept is assigned to at least one of the 134 semantic types. We only chose the terminologies mapped to health-related semantic types that we used in our previous study [36]. To obtain these medical terminologies automatically, we used MetaMap 2014 (US National Library of Medicine), a highly configurable program that maps biomedical text to concepts in the UMLS Metathesaurus. Using a Java application programming interface offered by MetaMap, we could parse the messages to get the health-related terminologies.

We performed clustering and keyword extraction for topic identification in the same manner as stakeholder identification.

#### Sentiment Analysis

We analyzed sentiment to assess the valence and intensity of the messages by community members. Lexicon-based approaches are widely used in sentiment analysis, and some well-known sentiment lexicons, such as SentiWordNet [37], have been successfully applied in sentiment analysis [38,39]. The SentiWordNet lexicon provides positive- and negative-intensity scores for each sentiment term. In this study, we used the SentiWordNet lexicon to extract sentiment terms from the messages and calculate their sentiment scores. We used the following 3 sentiment measures to evaluate sentiment expression: PositiveScores, NegativeScores, and SubjectiveScores [40,41], where PositiveScores is positive-polarity scores divided by the number of messages; NegativeScores is negative-polarity scores divided by the number of messages; and SubjectiveScores is subjective-polarity scores divided by the number of messages.

## Results

### Stakeholder Analysis

We used the expectation maximization clustering algorithm for stakeholder identification. Expectation maximization clustering can evaluate and determine the optimal number of clusters by cross-validating different numbers of clusters. We identified 3 stakeholder groups in 3 disease forums. We then performed the 2-sample *t* test to evaluate whether there was a significant difference in the 3 stakeholder groups. The null hypothesis was that the difference between 2 groups was 0. The conclusion was to reject the null hypothesis (*P*<.001) and that the 3 stakeholder groups were significantly different. Then, we extracted some keywords from each group according to equation (1) after filtering out meaningless or invalid phrases. Table 2 shows the results.

**Table 2:** Stakeholder analysis in the 3 disease forums.

Cluster	Keywords	Authorship
**Lung cancer**		
1	in my chest, on my chest, my lungs, my left lung, my right lung, please help me, of my chest, I was diagnosed, my ct scan, doctor tell me, my xray result, my question, i was wondering, I have cancer, be greatly appreciated	Patients
2	my husband, thank you, my mother, my dad, my mom, my sister, my question is, my father, do you think, i am worried, on his lung, thanks in advance, thanks so much, father in law	Caregivers
3	good luck, all the best, hope this helps, stay positive, you should, god bless, your mother, your father, your husband, with your doctor, you need to, let us know, sorry to hear, you could consider, see your doctor, your symptoms, with your physician, best of luck	Specialists
**Diabetes**		
1	my sugar, help me, I was diagnosed, my sugar levels, my blood, I was wondering, thank you, my body, my question, thanks for your, my blood sugar, I need to, my question is, I have diabetes, be greatly appreciated, type 1 diabetic	Patients
2	my husband, was diagnosed, he was diagnosed, her blood sugar, my son, my daughter, his blood sugar, low blood sugar, want to know, he has been, she has been, thanks so much, my son is, daughter was diagnosed	Caregivers
3	your blood, you need, your doctor, you need to, good luck, you should, your glucose, your blood sugar, I would suggest, your blood sugars, with your doctor, let us know, hope this helps, I hope you, your glucose levels,	Specialists
**Breast cancer**		
1	my breast, my nipple, my breasts, thank you, my question, should I, my left breast, in my right, on my left, in my breast, my right breast, found a lump, thanks for your, be greatly appreciated, of my breast	Patients
2	my mom, she had, my mother, her breast, she was diagnosed, family history, had breast cancer, my question is, worry about, her left breast, her right breast, my sister	Caregivers
3	I hope, you can, your doctor, you should, best wishes, your breast, good luck, you need to, your oncologist, let us know, with your doctor, hope this helps, all the best, a second opinion, second opinion	Specialists

Then, we could infer stakeholder identification according to the extracted keywords. Taking clustering results in the lung cancer forums as an example, in the cluster 1 group, members talked about their own conditions (eg, my chest, my lungs, my left lung, my right lung, of my chest, in my chest, on my chest) and procedures they underwent (eg, I was diagnosed, my xray result, my ct scan). They hoped to get help (eg, please help me, my question, i was wondering) and then gave thanks (eg, be greatly appreciated). We therefore assigned cluster 1 as the patient group. In the cluster 2 group, members talked more about their family members (eg, my husband, my mother, my father, my dad, my mom, my sister, father in law) and expressed their concern about their family members (eg, i am worried). They also raised some questions of concern (eg, my question is) and gave thanks (eg, thank you, thanks in advance, thanks so much). We assigned cluster 2 as the caregiver group. In the cluster 3 group, members were more likely to give other members advice and suggestions (eg, you should, you need to, you could consider) and offer help (eg, hope this helps) or advise them to undergo further consultation and procedures with their doctors (eg, with your doctor, see your doctor, with your physician). Members in the cluster 3 group often expressed their compassion and encouragement to other members (eg, stay positive, good luck, all the best, sorry to hear, best of luck). We thus assigned cluster 3 as the health specialist group.

Then we used the 3 evaluation metrics to test whether the textual features proposed in this study could significantly distinguish different stakeholders. By incorporating writing style-based features (F1), word *n*-grams (F2), medical domain-specific terminologies (F3), and kinship terminologies (F4) into the feature set in turn, we evaluated the results of clustering based on different feature sets, as Table 3 shows. The results showed that the 3 measure values increased as more features were incorporated into the feature set, and reached a maximum (eg, Rand=0.802, Jaccard=0.393, FM=0.537 for breast cancer) when all features were incorporated, indicating that these textual features could improve the performance of stakeholder identification significantly.

**Table 3:** Performance measures for distinguishing stakeholders using different textual feature sets^a^.

Disease	Feature set	Performance measure		
		Rand index	Jaccard similarity coefficient	Fowlkes-Mallows index
Lung cancer	F1	0.712	0.261	0.395
	F1+F2	0.731	0.321	0.441
	F1+F2+F3	0.757	0.349	0.473
	F1+F2+F3+F4	0.785	0.371	0.501
Diabetes	F1	0.717	0.273	0.401
	F1+F2	0.742	0.335	0.456
	F1+F2+F3	0.780	0.367	0.489
	F1+F2+F3+F4	0.792	0.381	0.523
Breast cancer	F1	0.725	0.297	0.421
	F1+F2	0.779	0.356	0.481
	F1+F2+F3	0.793	0.385	0.529
	F1+F2+F3+F4	0.802	0.393	0.537

^a^Feature set components: style-based features (F1), word *n*-grams (F2), medical domain-specific terminologies (F3), and kinship terminologies (F4).

**Figure 2:**
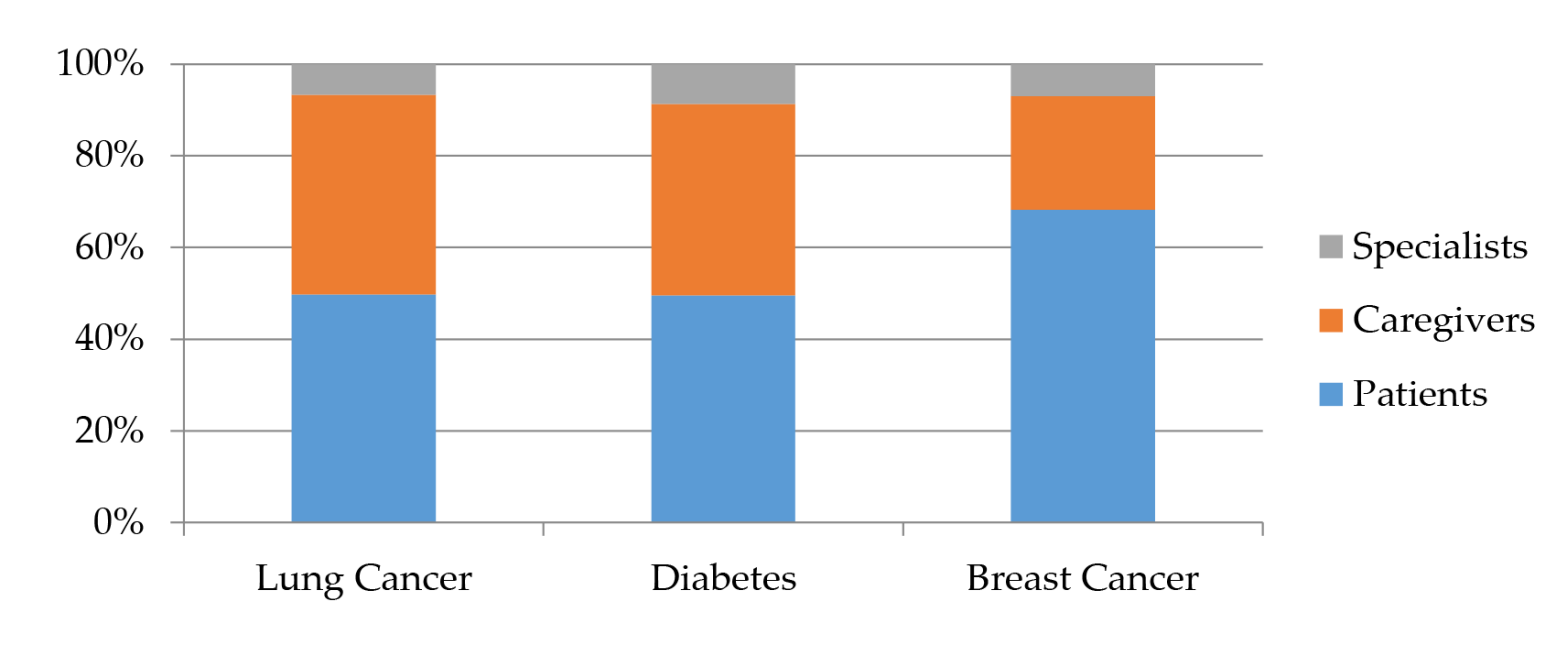
Distributions of the 3 stakeholder groups.

In terms of the distributions of different stakeholders in the 3 disease forums, as Figure 2 shows, patients (24,429/39,606, 61.68%) and caregivers (12,232/39,606, 30.88%) were the majority of the population, in contrast to health specialists (2945/39,606, 7.43%). The proportions of patients (1202/2416, 49.75% vs 5738/11,571, 49.59%) and caregivers (1053/2416, 43.58% vs 4836/11,571, 41.79%) were similar in the lung cancer and diabetes forums. By contrast, in the breast cancer forum, the proportion of patients (17,489/25,619, 68.27%) was significantly greater.

The reason for the different distributions may be that women are the prominent group with breast cancer and are also more likely than men to seek online information, as indicated by a previous study [42].

Table 4 shows descriptive statistics of postings by different stakeholders. Both patients and caregivers published fewer than 2 messages (1.48 and 1.51) on average. By contrast, health specialists, although constituting a minority of the population in the online community compared with patients and caregivers, published many more postings (28.4) on average than the other 2 groups.

We speculated about the reason for different distributions of posting. Generally, most patients and caregivers aimed to seek health information they were concerned about. They would leave the site after receiving satisfactory answers. Another possibility was that they simply became frustrated with the inability to find answers and left the site soon. Most patients and caregivers thus were short-term participants.

**Table 4:** Clustering results in the stakeholder analysis.

Disease	Cluster	No. of members	No. of messages	Messages per member	Authorship
Lung cancer	1	1202	1378	1.15	Patients
	2	1053	1607	1.53	Caregivers
	3	161	2332	14.48	Specialists
Diabetes	1	5738	7691	1.34	Patients
	2	4836	7136	1.48	Caregivers
	3	997	20,366	20.43	Specialists
Breast cancer	1	17,489	27,012	1.55	Patients
	2	6343	9727	1.53	Caregivers
	3	1787	60,912	34.09	Specialists

**Figure 3:**
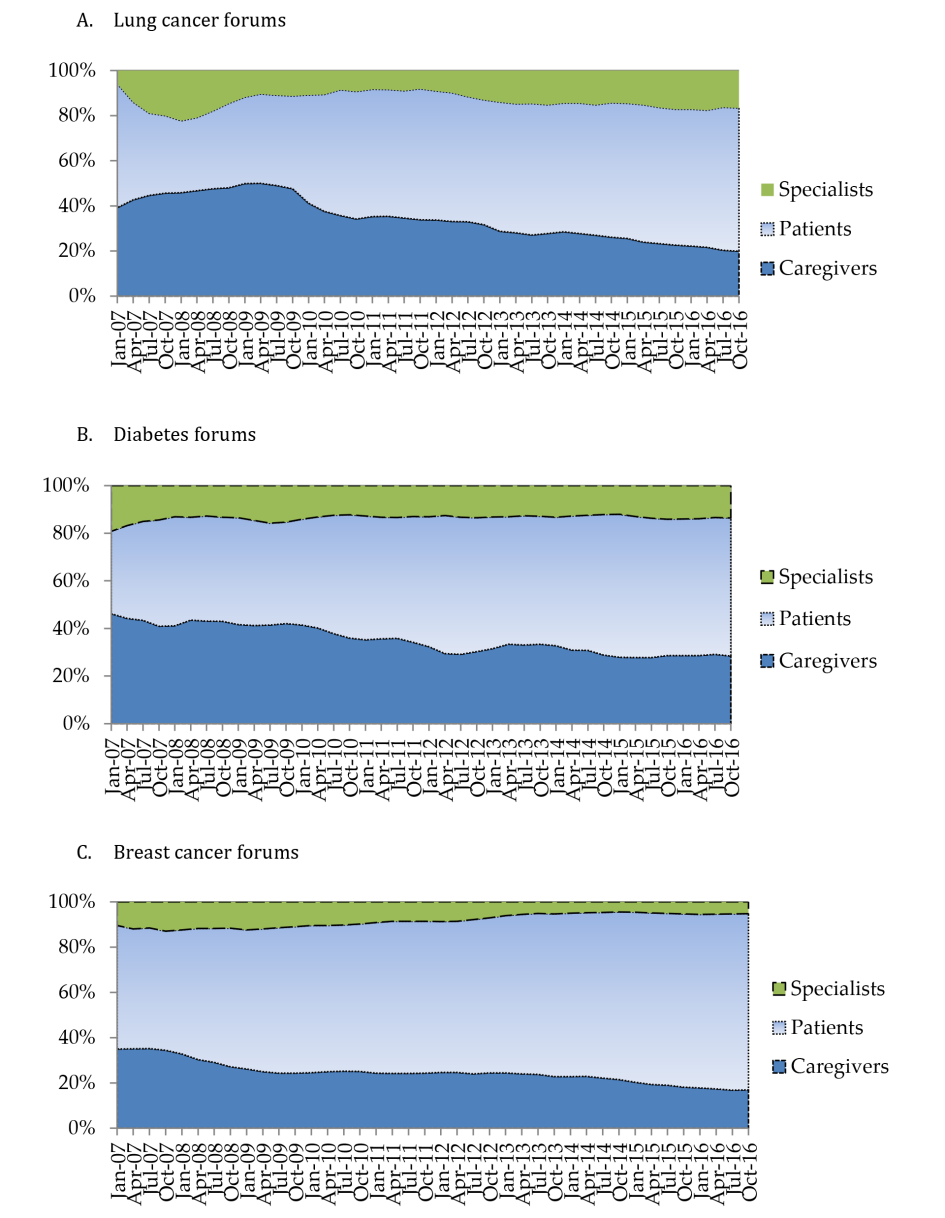
Changes in the proportions of stakeholder groups in (A) lung cancer, (B) diabetes, and (C) breast cancer forums, January 2007 to October 2016.

In terms of changes in the proportions of the 3 stakeholder groups (see Figure 3), the proportion of the specialist group tended to decrease and then stabilize. By contrast, the proportion of caregivers decreased gradually, and the proportion of patients increased.

One possible explanation is that most health specialists gradually formed a stable relationship with the online community and only a few left after short-term engagement. By contrast, as health care social media becomes more widespread and widely accepted, a higher proportion of patients than of caregivers prefer to seek online health information. After all, patients themselves are most concerned about their own health conditions.

### Topic Analysis

Clustering and keyword extraction for topic identification were performed in the same manner as stakeholder identification. We identified 5 significantly different health hot topics (*P*<.001), as Table 5 shows. They were named based on the extracted keywords with similar UMLS semantic types: symptom, examination, procedure, drug, and complication.

**Table 5:** Topic analysis in the 3 disease forums.

Cluster	Topics	Keywords	UMLS^a^ semantic types
**Lung cancer**			
1	Symptom	cough, pain, breathless, symptoms, chest pain, painful, shortness of breath, wheezing, short of breath, coughing up blood, nausea	sosy
2	Complication	infection, bronchitis, pneumonia, tuberculosis, asthma, pleural effusion, copd, emphysema, collapsed lung, atelectasis	dsyn, patf
3	Examination	scans, x-ray, cat scan, mri, biopsy, pet scan, chest x-ray, imaging, biopsy needle, bronchoscopy	diap
4	Procedure	chemo, operation, surgery, radiation, therapy, chemotherapy, removal, radiation therapy, wedge resection, lobectomy	topp
5	Drug	silicas, morphine, advil, tarceva, chantix, carboplatin, alimta, dilaudid, taxol, coumadin	phsu
**Diabetes**			
1	Drug	insulin, lantus, januvia, metformin, glucophage, actos, avandia, amaryl, marijuana, glipizide	phsu
2	Complication	infection, hypoglycemia, low blood sugar, dka, obesity, pcos, kidney disease, coma, diabetic neuropathy, bgs	dsyn, patf
3	Symptom	pain, tired, thirsty, nausea, fatigue, frequent urination, hungry, dizzy, itchy, sore, tingling	sosy
4	Examination	blood test, glucose test, fasting test, fasting blood sugar, cat scan, hemoglobin a1c test, gtts, glucose tolerance test, mri	lbpr, diap
5	Procedure	infusion, therapy, injection, transplant, dialysis, rx, ect, insulin injection, cde, amputation	topp
**Breast cancer**			
1	Examination	biopsy, mri, ultrasound, mammogram, screening, bi-rads, core biopsy, cat scan, imaging, biopsy needle	diap, lbpr
2	Procedure	chemo, operation, chemotherapy, radiation, radiotherapy, mastectomy, lumpectomy, implant, removal, surgical	topp
3	Symptom	sore, pain, painful, breast pain, nipple discharge, itching, tingling, hot flashes, nausea, itchy	sosy
4	Drug	tamoxifen, arimidex, taxol, femara, taxotere, carboplatin, effexor, docetaxel, valium, raloxifene	phsu
5	Complication	infection, rash, lymph edema, fibrocystic breast, mastitis, idc, eczema, complex cyst, complex cysts, neuropathy, fibrocystic breast disease, fibrocystic disease	dsyn

^a^UMLS: Unified Medical Language System.

We then used the 3 evaluation metrics to test whether the textual features proposed in this study could be used to distinguish different hot topics significantly. By incorporating word *n*-grams (F1) and medical domain-specific terminologies (F2) into the feature set, we evaluated the results of clustering based on a 2-feature set, as s Table 6 shows. The results showed that feature set F1+F2 outperformed F1 significantly (eg, Rand=0.783, Jaccard=0.369, FM=0.495 for breast cancer), indicating that the 2 types of textual features improved the performance of topic identification significantly.

**Table 6:** Performance measures for distinguishing hot topics using different textual feature sets^a^.

Disease	Feature set	Rand	Jaccard	FM^b^
Lung cancer	F1	0.703	0.242	0.382
	F1+F2	0.761	0.352	0.478
Diabetes	F1	0.718	0.275	0.411
	F1+F2	0.774	0.351	0.478
Breast cancer	F1	0.722	0.285	0.417
	F1+F2	0.783	0.369	0.495

^a^Feature set components: word *n*-grams (F1) and medical domain-specific terminologies (F2).

^b^FM: Fowlkes-Mallows index.

**Figure 4:**
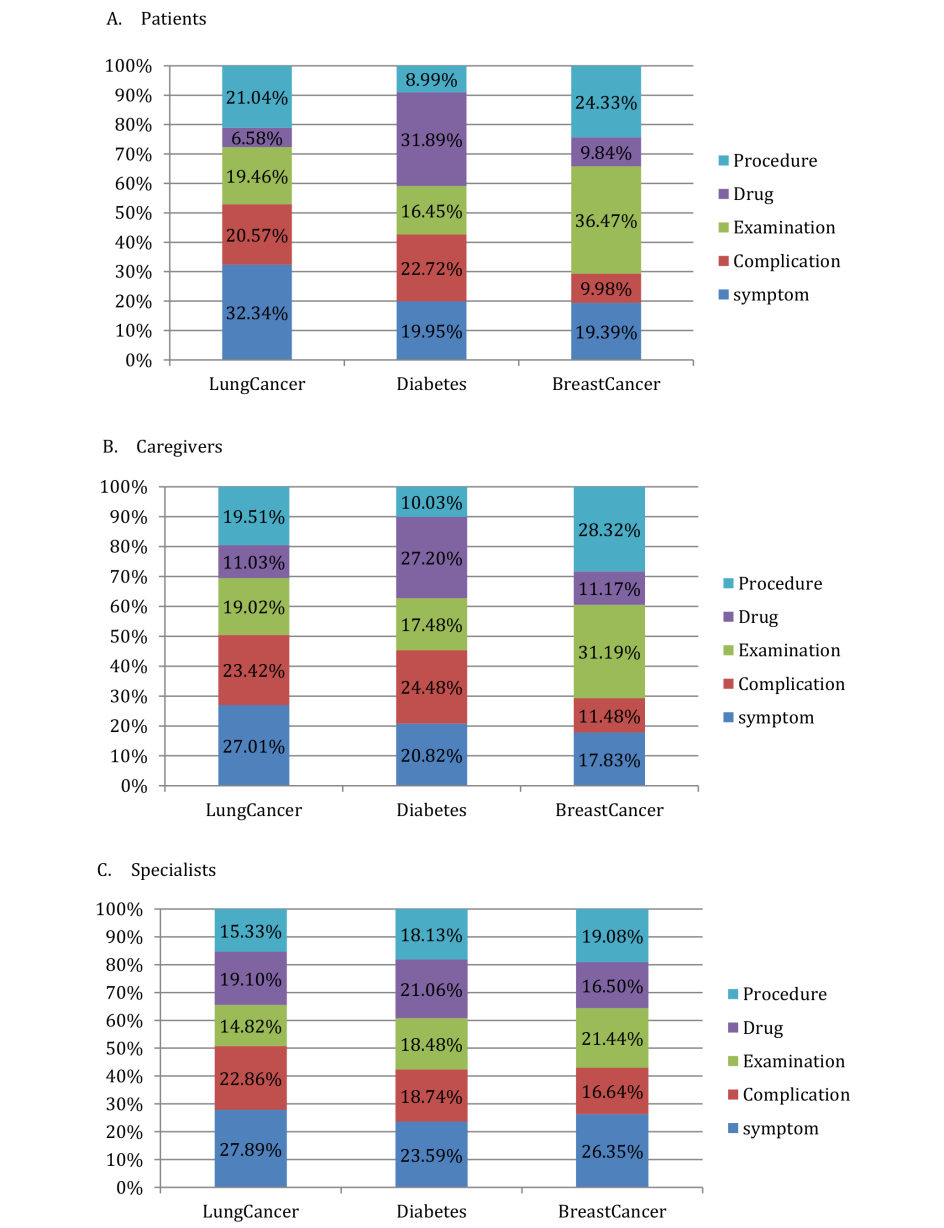
Distributions of (A) patients, (B) caregivers, and (C) specialists in the 5 hot topics.

To further explore the concerns of different stakeholders, we examined the distributions of the 3 stakeholder groups in the 5 hot topics (Figure 4). Among the different stakeholders in the different types of diseases, their concerns about various health topics were fairly different.

A significantly greater proportion of patients with lung cancer (536/1657, 32.34%) were involved in the symptom topics. Patients with diabetes were more interested in drug topics (1883/5904, 31.89%). Patients with breast cancer were more likely to mention their examination or tests online (8728/23,934, 36.47%).

The reasons may be that some early symptoms of lung cancer, such as coughing or wheezing, are very similar to ailments such as fevers and bronchitis. Patients with such ailments were not sure whether their conditions were signs of lung cancer and thus hoped to obtain valuable information by seeking the experiences of others or consulting about their conditions on health care social media. By contrast, patients who found an abnormality of their breast tissues usually underwent further examination. Because of a lack of other obvious symptoms, they mainly discussed their examination or tests for the diagnosis of breast cancer. Diabetes is a common chronic disease that is difficult to cure. Patients with diabetes use regular long-term medication and thus were naturally concerned about topics related to antidiabetic drugs to learn about their medical effects and possible side effects.

The distribution of hot topics among caregivers was similar to that of patients. Both groups appeared to be mainly concerned with the hottest health issues related to a specific disease, as patients and caregivers were both information requesters. However, the proportional distribution of topics was distinguishable between patients and caregivers. For example, a significantly greater proportion of caregivers than of patients were involved in drug topics (300/2721, 11.03% for caregivers vs 109/1657, 6.58% for patients) in lung cancer forums and in procedure topics (3952/13,954, 28.32% for caregivers vs 5822/23,934, 24.33% for patients) in breast cancer forums.

These results indicate that caregivers were more concerned about disease treatment topics than disease diagnosis because drug and procedure topics are both related to disease treatment. Complication was another health topic of interest to caregivers, possibly because it may greatly affect their caregiving responsibilities. As Figure 4 shows, the proportion of complication topics was significantly greater among caregivers than among patients in the 3 disease forums (4449/25,701, 17.31% for caregivers vs 4070/31,495, 12.92% for patients).

As Figure 4 shows, health specialists were equally distributed among the 5 health topics, in contrast to patients and caregivers. This result indicates that specialists, as information providers, were more likely to focus on various health topics and to share their experiences and knowledge. Broad knowledge is indispensable for health specialists to offer help to patients in their time of need.

### Sentiment Analysis

**Figure 5:**
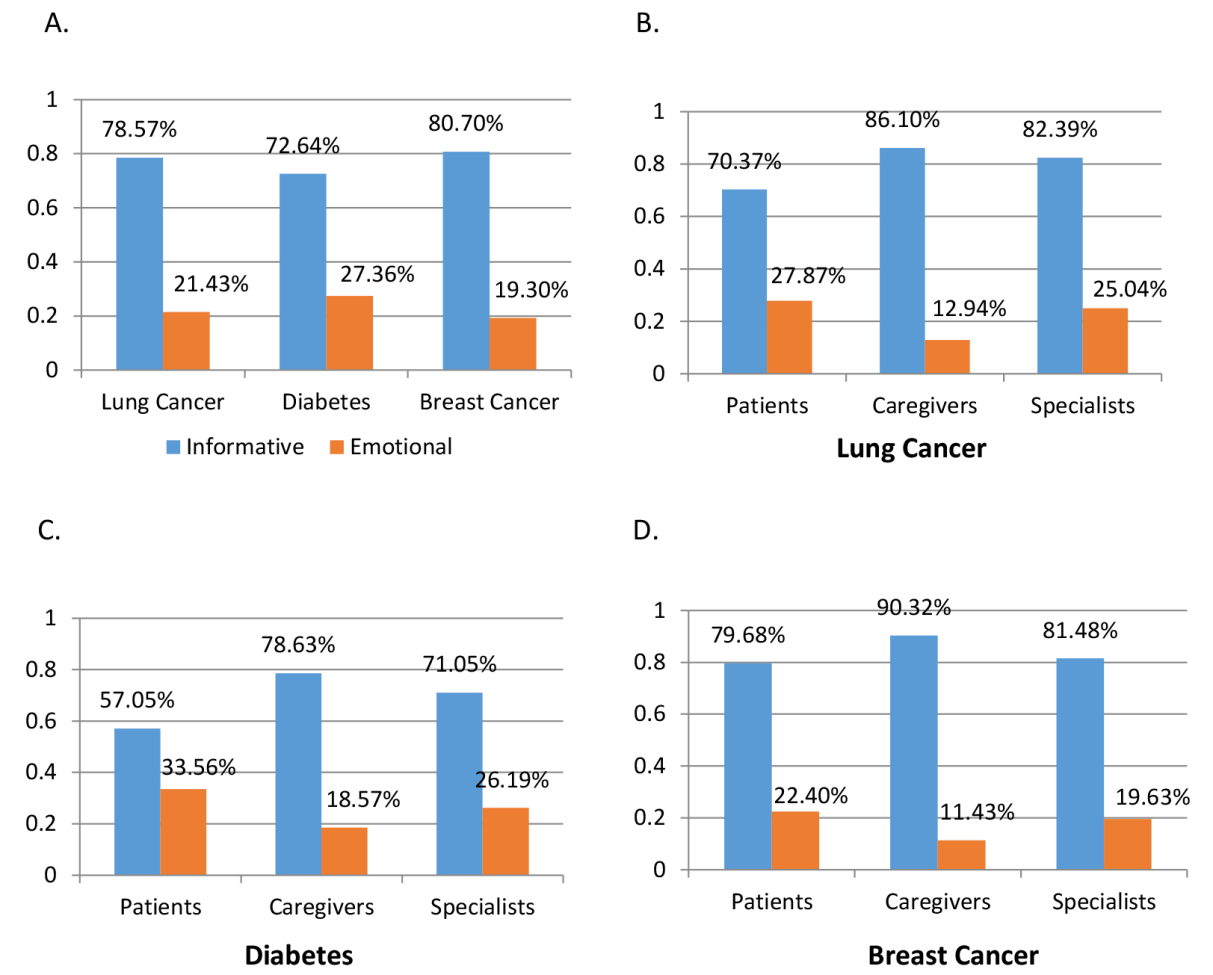
Distributions of informative messages and emotional messages by (A) disease and by stakeholder group in (B) lung cancer, (C) diabetes, and (D) breast cancer forums.

Emotional messages could be distinguished from informative messages by setting a threshold of sentiment measures in SubjectiveScores. As Figure 5 shows, informative messages (108,519/138,161, 78.54%) were more prevalent than emotional messages (29,642/138,161, 21.45%).

This result suggests that most users make use of health care social media more to exchange health information than to provide a platform for emotional communication. This result is also in agreement with the above argument that most patients and caregivers were short-term participants and that many only sought health information of interest.

The distribution of informative and emotional messages among different stakeholders indicated that patients were more likely to express their emotions than caregivers and specialists. The proportion of emotional messages was 25.05% (9040/36,081) for patients, 14.39% (2659/18,470) for caregivers, and 21.46% (17,943/83,610) for specialists. Particularly among patients in the diabetes forums, patients usually encouraged each other to overcome their diseases by pursuing long-term treatment. By contrast, caregivers preferred to share informative messages and were the least likely to exchange emotions among the 3 stakeholders.

**Figure 6:**
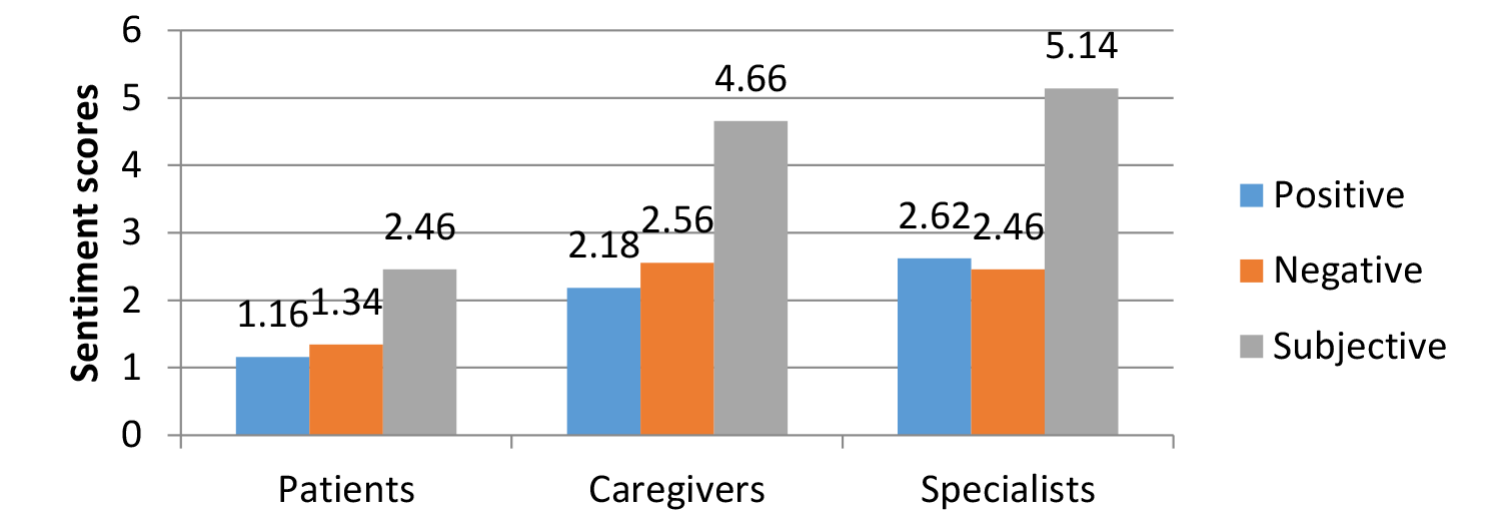
Sentiment measures of the 3 stakeholder groups.

One possibility is that the caregiving responsibilities of caregivers do not leave sufficient time for online communication. Especially for the caregivers involved in the 2 cancer forums, most only sought diagnosis and treatment information of interest online to help their patients cure their high-survival-rate diseases.

We calculated the 3 sentiment measures of PositiveScores, NegativeScores, and SubjectiveScores to evaluate sentiment expression, as Figure 6 shows. The subjective sentiment scores of patients (2.46) were lower than those of caregivers (4.66) and specialists (5.14), indicating that, although patients were more likely to express their emotions, as Figure 5 shows, the emotional intensity of patients was lower than that of specialists and caregivers. The negative sentiment scores of caregivers (2.56) were higher than their positive scores (2.18). Among health specialists, positive sentiment scores (2.62) were higher than their negative sentiment scores (2.46).

We speculated about the reasons for the differences in sentiment expressions. Patients appear to prefer to regard health care social media as an emotion-exchange platform rather than a place to vent their negative feelings. Conversely, caregivers undertaking long-term caretaking activities usually expressed strong emotions about their patients. They preferred to use negative words with high emotional intensity to vent their emotional stress and burden [43,44], as Figure 6 shows that the negative sentiment scores of caregivers were higher than their positive scores. Many caregivers appeared to regard health care social media as a good platform to vent and relieve stress. For health specialists, higher positive sentiment scores implied that this group provided emotional support by encouraging patients and their caregivers to confront their diseases rather than expressing sympathy and pity.

**Figure 7:**
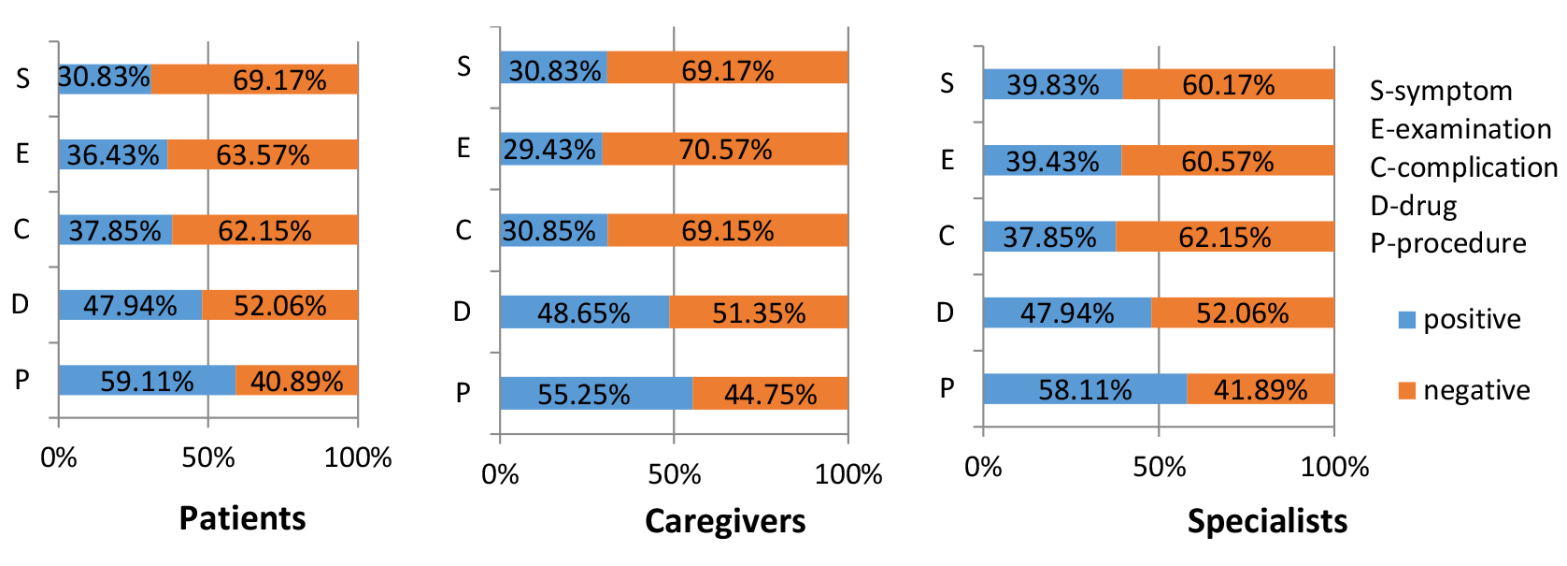
Distributions of positive and negative messages posted by the 3 stakeholder groups in the 5 hot topics.

Further analysis identified differences in sentiment expression by different stakeholders in different hot topics, as Figure 7 shows. Overall, for patients and caregivers, the proportions of negative emotional messages were greater than those of positive emotional messages among the topics related to symptom, complication, and examination; for example, for patients, the proportion was 30.83% (562/1823) positive versus 69.17% (1261/1823) negative in symptom topics, 36.43% (1055/2896) versus 63.57% (1841/2896) in examination topics, and 37.85% (450/1189) versus 62.15% (739/1189) in complication topics.

This result indicates that patients and caregivers who discussed complications and examination topics feared illness, depression, hopelessness, anxiety, and other negative emotions and thus were more likely to express negative emotions. In particular, among caregivers, negative emotional intensity was significantly higher for the complication topic than for the other topics, indicating that complications were a considerable burden for caregivers performing long-term caretaking activities, and this group expressed strong negative feelings of disappointment and even despair. By contrast, drug and procedure topics are usually more closely related to the treatment of disease, and thus all stakeholders who discussed these 2 topics were more likely to have a positive attitude; for example, for patients, the proportion was 47.94% (580/1210) positive versus 52.06% (630/1210) negative in drug topics and 59.11% (1136/1922) versus 40.89% (786/1922) in procedure topics. In their discussion of the 2 topics, patients were happy to be getting better, and the caregivers expressed their satisfaction with the treatment; in addition, specialists conveyed their congratulations and best wishes to the patients.

**Figure 8:**
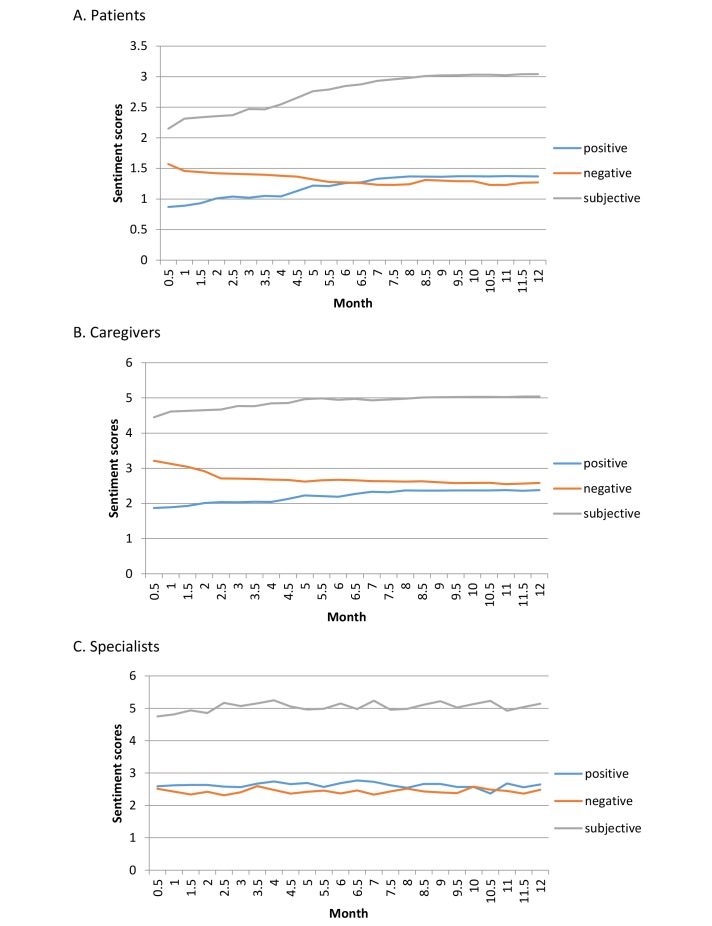
Changes in the sentiment expression of (A) patients, (B) caregivers, and (C) specialists over the first year of each user’s involvement.

Finally, we analyzed trends in emotional changes among the different stakeholders to illustrate whether members who engaged in health care social media changed their sentiment expression based on online interactions with other users. We incorporated the messages posted by different members every half month and calculated the changes in the 3 sentiment measures of PositiveScores, NegativeScores, and SubjectiveScores over time in the first year of their involvement. Figure 8 shows the results.

As Figure 8 shows, the subjective sentiment scores of the messages from patients tended to increase in the early weeks and then stabilized. Patients preferred to express concern about their health topics of interest during their early involvement and then gradually began to express their feelings for social support. From the perspective of sentiment polarity, we found that positive sentiment scores increased gradually while negative sentiment scores decreased, implying that the patients engaged in health care social media changed their emotional state in a positive direction through online interactions with other users. Some possible reasons for this change are that the patients eliminated fears and anxiety after their problems were resolved or that professionals and fellow patients encouraged them to confront their diseases.

The subjective sentiment scores of the messages from caregivers remained high. Caregivers apparently preferred to use subjective words with high emotional intensity in their posts compared with patients. From the perspective of sentiment polarity, positive sentiment scores increased gradually while negative sentiment scores decreased, similar to the trend changes in patients, possibly for the same reasons discussed above. However, in contrast to the sentiment expression of patients, for caregivers the negative sentiment scores remained higher than the positive sentiment scores, implying that caregivers appeared to regard health care social media as a platform to share their negative feelings. Caregivers may complain about their long-term, heavy caretaking work online to relieve their stress at any time.

All 3 sentiment scores of the messages from specialists remained stable over time. This result indicates that specialists were involved long term in the online community to provide emotional support and to help patients and caregivers in various ways. They expressed their sympathy and pity for the unfortunate users by using some negative words while encouraging them to fight their illness using some positive words.

## Discussion

Understanding the concerns of different stakeholders and how they use social media is very meaningful work. In this paper, we proposed a novel framework using text mining techniques to perform a comprehensive content analysis of an online health community from the perspectives of diverse stakeholders. We used a computational social science approach to process the large amount of text data for stakeholder analysis, topic analysis, and sentiment analysis. We identified significant differences in hot topics of interest and sentiment expression among different stakeholders involved in different types of disease forums. These valuable conclusions provide a better understanding of health care social media use by different stakeholders that may aid improvements in social media services to facilitate diverse stakeholder engagement for more effective health information sharing and social interaction.

This study also has some limitations that must be considered further. Further research should examine how to describe and measure the impacts of health care social media use on health self-management. Users involved in social media maintained good communication and developed online social networks, and thus future studies should include social network analysis of different stakeholder groups to determine the impacts of these relationships on their engagement. In addition, some deeper stakeholder analysis by using text mining techniques, such as how to distinguish knowledgeable patients and professional doctors in specialist groups, would be considered in our further study.

## Abbreviations

FM: Fowlkes-Mallows.
MeSH: Medical Subject Headings
UMLS: Unified Medical Language System
